# Predictors of COVID-19 Vaccine Acceptance, Intention, and Hesitancy: A Scoping Review

**DOI:** 10.3389/fpubh.2021.698111

**Published:** 2021-08-13

**Authors:** Ashish Joshi, Mahima Kaur, Ritika Kaur, Ashoo Grover, Denis Nash, Ayman El-Mohandes

**Affiliations:** ^1^City University of New York Graduate School of Public Health and Health Policy, New York, NY, United States; ^2^Foundation of Healthcare Technologies Society, New Delhi, India; ^3^Indian Council of Medical Research, New Delhi, India

**Keywords:** COVID-19 vaccine, vaccine surveys, vaccine acceptance, vaccine hesitancy, vaccine rate

## Abstract

COVID-19 vaccine is regarded as the most promising means of limiting the spread of or eliminating the pandemic. The success of this strategy will rely on the rate of vaccine acceptance globally. The study aims to examine the factors that influence COVID-19 vaccine acceptance, intention, and hesitancy. PubMed was searched comprehensively for articles using the keyword “COVID-19 vaccine surveys.” Of the 192 records, 22 studies were eligible for the review. Eighty-two percent of these studies were conducted among the general population. Gender, age, education, and occupation were some of the socio-demographic variables associated with vaccine acceptance. Variables such as trust in authorities, risk perception of COVID-19 infection, vaccine efficacy, current or previous influenza vaccination, and vaccine safety affected vaccine acceptance. Globally, in March 2020, the average vaccine acceptance observed was 86% which dropped to 54% in July 2020 which later increased to 72% in September 2020. Globally, the average rate of vaccine hesitancy in April 2020 was 21%, which increased to 36% in July 2020 and later declined to 16% in October 2020. Large variability in vaccine acceptance and high vaccine hesitancy can influence the efforts to eliminate the COVID-19. Addressing the barriers and facilitators of vaccines will be crucial in implementing effective and tailored interventions to attain maximum vaccine coverage.

## Introduction

The COVID-19 pandemic continues to impose enormous burdens on morbidity and mortality while severely disrupting societies and economies worldwide. Governments prepare themselves to ensure large-scale, equitable access and distribution of safe and effective COVID-19 vaccines. Overcoming the pandemic will require sufficient health system capacity, and effective strategies to enhance trust in and acceptance of vaccines. Concern about vaccine hesitancy is growing worldwide (1). For decades, vaccines have been a successful measure to eliminate and prevent numerous infections. However, vaccine hesitancy and misinformation act as hurdles in achieving high coverage and community immunity against the infection (2, 3). In 2015, the World Health Organization (WHO) Strategic Advisory Group of Experts on Immunization stated vaccine hesitancy as a “delay in acceptance or refusal of vaccination despite the availability of vaccination services” (4). Vaccine hesitancy can differ in form and intensity based on when and where it occurs and what vaccine is involved (5, 6). Concerns about vaccine hesitancy are growing globally, prompting the World Health Organization (WHO) to declare it among the top ten health threats in 2019 (7).

Governments, public health officials, and advocacy groups must be equipped to address vaccine hesitancy. There is a need to build vaccine literacy to increase vaccine acceptance rates. Besides, misinformation spread through multiple sources could have a considerable impact on the acceptance of a COVID-19 vaccine (8). Governments and societies must gauge current levels of willingness to receive potentially safe and effective COVID-19 vaccines and identify correlates of vaccine hesitancy and/or acceptance. Intervention models to improve vaccine literacy and acceptance should directly take up community-specific concerns, misconceptions, and be sensitive to religious or cultural beliefs (9). Researchers have recognized effective interventions for building confidence and decreasing vaccine hesitancy in different contexts (10, 11). Trust in government is highly associated with vaccine acceptance and can contribute to public compliance with recommended actions (12). Addressing and overcoming vaccine hesitancy requires more than building trust. Clear and consistent effective communication by government officials is central in building public confidence in vaccine programs. This includes explaining how vaccines work, their development, along regulatory approval based on safety and efficacy. Powerful campaigns should also aim to explain the effectiveness of vaccines, the time needed for protection, and the significance of population-wide vaccine coverage to attain community immunity. Inculcating public confidence in regulatory agency reviews of vaccine safety and effectiveness will be imperative (13, 14). Despite tremendous efforts being made to achieve COVID-19 vaccine coverage, vaccine hesitancy could be a major barrier toward its acceptance by the general population. To identify the scope of the problem, the current scoping review aims to explore and understand the rates of acceptance and hesitancy related to COVID-19 vaccine among the population globally. This could help bridge the knowledge gaps and facilitate formation of effective strategies to overcome the high levels of hesitancy related to COVID-19 vaccine, increase its uptake, and mitigate the pandemic as well as help global stakeholders to conduct COVID-19 vaccination drives and promote vaccine uptake.

## Research Objectives

The objectives of the review include:

1. To examine the factors that influence COVID-19 vaccine acceptance, intention, and hesitancy using findings of the various COVID-19 vaccine surveys conducted globally.
2. To develop a conceptual framework of factors that influence COVID-19 vaccine acceptance, intention, and hesitancy globally.
3. To explore and assess the rate of COVID-19 vaccine acceptance, intention, and hesitancy globally.

## Methodology

### Search Strategy

**Figure 1:**
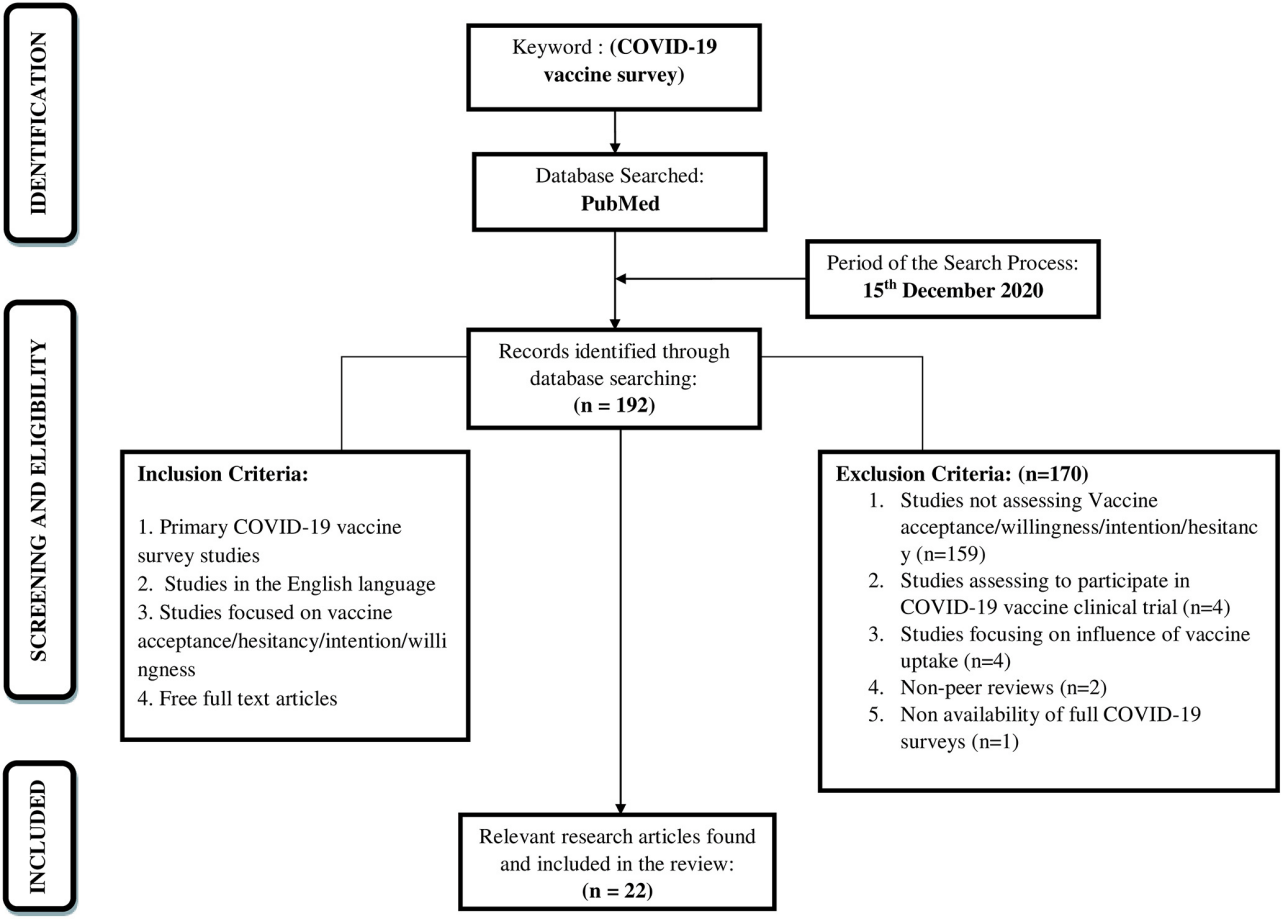
Flowchart of the search strategy and the study selection.

A literature search was conducted on 15th December 2020 to have a comprehensive understanding of the focussed research topic. The review would intend to identify the available research literature that would aid in evidence-based practice (15). The current scoping review adopted an iterative Five-stage methodological framework comprising of the following steps: (i) identification of research question, (ii) identification of relevant research articles, (iii) study selection, (iv) charting the data, (v) collating, reporting, and summarizing the findings (16). The database searched, search strategy, eligibility criteria, and the selection of studies were described. The search for articles was reported using Preferred Reporting Items for Systematic reviews and Meta-Analysis (PRISMA) extension for Scoping Reviews (PRISMA-ScR) Checklist (Figure 1). A literature search was carried out in the “PubMed” research database as it is an authentic and reliable source for conducting research and indexing the research articles. Reference lists of the included studies and reviews checked for additional studies of relevance to the review (backward reference list checking).

### Search Terms

The keywords used for searching in the database included (COVID-19 vaccine survey). The search terms were derived from previous reviews and informatics experts interested in vaccine research.

The advanced search detail for the keyword is given below:

(“covid 19 vaccines”[MeSH Terms] OR (“covid 19”[All Fields] AND “vaccines”[All Fields]) OR “covid 19 vaccines”[All Fields] OR “covid 19 vaccine”[All Fields]) AND (“survey s”[All Fields] OR “surveyed”[All Fields] OR “surveying”[All Fields] OR “surveys and questionnaires”[MeSH Terms] OR (“surveys”[All Fields] AND “questionnaires”[All Fields]) OR “surveys and questionnaires”[All Fields] OR “survey”[All Fields] OR “surveys”[All Fields])

### Study Eligibility Criteria

Inclusion and exclusion criteria of the research articles utilized during the electronic search, to guide the selection of the research articles as summarized below. Original studies focusing on vaccine acceptance, willingness, hesitancy, or intention regarding the COVID-19 vaccine were included. Studies were excluded from the scoping review if they were not assessing Vaccine acceptance/willingness/intention/hesitancy, were assessing to participate in COVID-19 vaccine clinical trial, studies focusing on influence of vaccine uptake, Non-peer review studies, and if there was no availability of full COVID-19 survey.

The current review followed two steps in selecting the studies. In the first step, two reviewers (RK & MK) screened independently the titles and abstracts of all retrieved studies. In the second step, the same reviewers read independently the full texts of studies included from the first step. Any disagreements between both reviewers were resolved through consulting a third reviewer (AJ).

### Data Extraction

To conduct a systematic and accurate extraction of data, a data extraction form was developed, and similar to the study selection process, two reviewers (MK and RK) independently conducted the process of data extraction, and any disagreements were resolved by the third reviewer (AJ).

### Study Quality Assessment

Scoping reviews are different from systematic reviews as they include broader topics and include studies with more diverse study designs. Therefore, scoping reviews usually do not focus on the quality assessment of the included studies. Accordingly, the quality assessment of the included studies was not performed in the review (15).

### Data Synthesis

Extracted data were synthesized using a narrative approach (17). The authors of the study attempted to classify the COVID-19 vaccine survey studies according to the following variables to be included in the final analysis;

**Time of Survey:** Information gathered on the date and duration of the surveys conducted.

**Study country and location**: Information recorded in the country where the vaccination survey was conducted and whether or not the surveys are done at a global or national level.

**Study Objectives:** Information recorded to assess whether the study aimed to assess vaccine acceptance, vaccine hesitancy, and vaccine intention or vaccine determinants.

**Study Design:** Information was recorded on whether the studies were cross-sectional studies or not.

**Sample Size:** Information on the total number of respondents enrolled in each study extracted.

**Survey Platform:** Information recorded on the platform used to gather the primary data and the language in which the survey was disseminated.

**Survey Instruments:** Information was recorded on the variables included in the vaccine survey questionnaire by the studies.

**Outcome Assessed:** It included the prevalence of vaccine acceptance, vaccine hesitancy, and vaccine determinants.

**Limitations:** Limitations of each study were assessed so that areas of further research were identified.

### Data Analysis

Characteristics of studies were summarized in tables and described narratively. Then, a description of the characteristics of vaccine hesitancy, acceptance, and its determinants in the included studies was presented.

## Results

### Search Results

Using the above-mentioned selection criterion, the electronic search yielded 192 research articles on 15th December 2020 (Figure 1). The identified research articles were examined based on the titles and abstracts for additional relevant research articles. Further, the search terms and reference lists provided in the relevant identified research articles were utilized to develop a search strategy and identify further relevant research articles through forward and backward search. Following this, 22 research articles were found to fulfill the inclusion criteria and were included in the review (Supplementary Table 1). Thus, the full texts of selected research articles were reviewed based on inclusion and exclusion criteria by the authors of the present study.

### Timeline Brief

**Figure 2:**
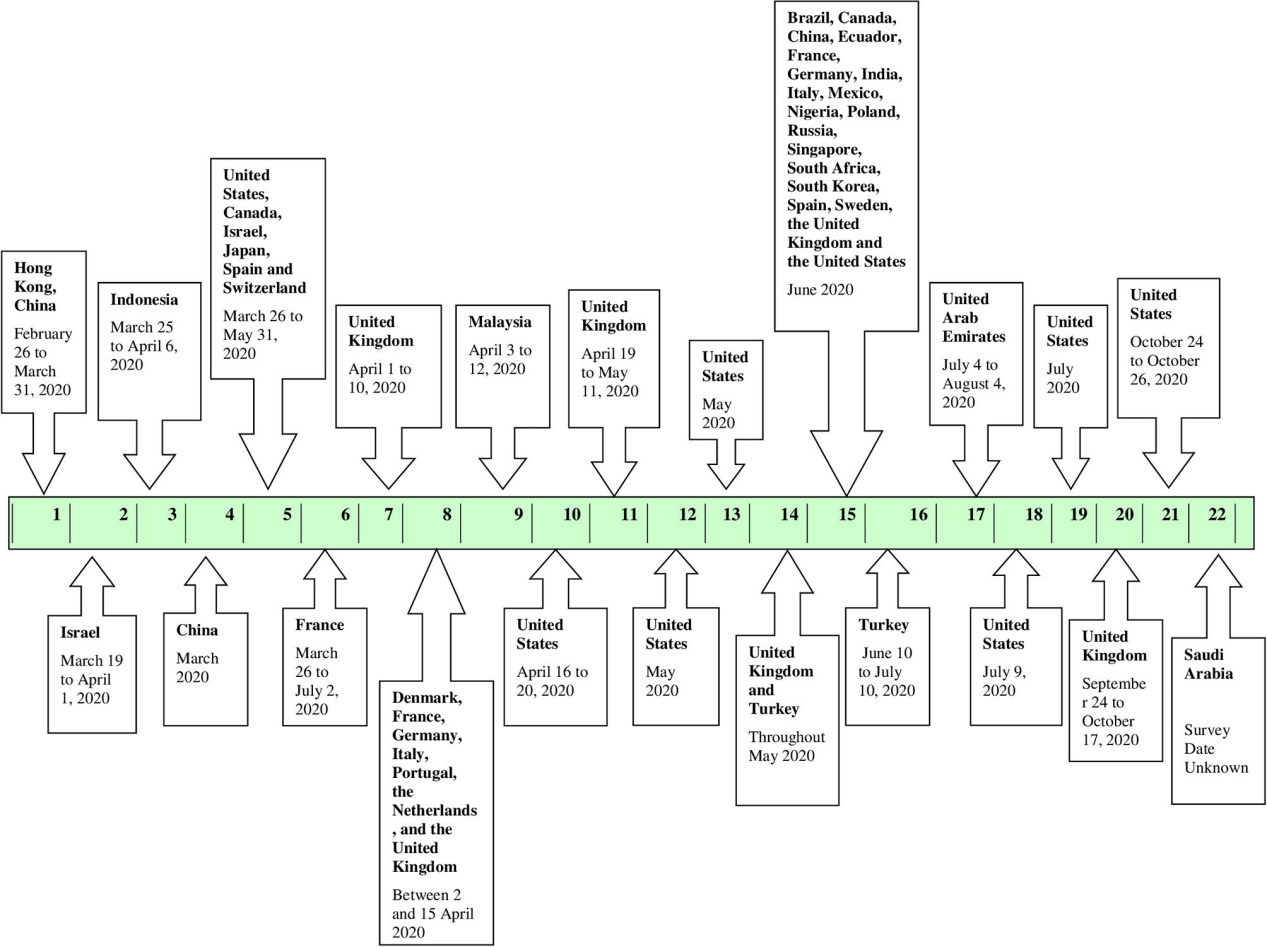
Timeline of the COVID-19 vaccine surveys conducted globally.

Figure 2 depicts the start date/month of the vaccine surveys conducted in the various countries. From the eligible 22 studies analyzed, the first vaccine survey was conducted in Hong Kong, China among nurses on 26 February and 31 March 2020 (18). The second survey was conducted over 2 weeks, starting March 2020, 1 week after initiating social distancing and quarantine regulations by the government, in Israel (19). Four more vaccine surveys were initiated in March 2020, one of which was conducted at a global level (20–23). Three of the four studies were conducted in Israel, Indonesia, and China at the national level, and one was a global study conducted in the United States, Canada, Israel, Japan, Spain, and Switzerland (19–22). In April 2020, another vaccine survey was conducted between 1st April and 10th April 2020 in the United Kingdom (24). A global study was conducted in Denmark, France, Germany, Italy, Portugal, the Netherlands, and the United Kingdom between 2 and 15 April 2020 (25). Following this, three more vaccine surveys commenced in Malaysia, the United States, and United Kingdom (26–28). Further, in May 2020, two more vaccine surveys were conducted in the United States followed by a vaccine survey conducted in two countries, namely, the United Kingdom and Turkey (29–31). In June 2020, a global vaccine survey was conducted in 19 countries namely, Brazil, Canada, China, Ecuador, France, Germany, India, Italy, Mexico, Nigeria, Poland, Russia, Singapore, South Africa, South Korea, Spain, Sweden, the United Kingdom, and the United States followed by a national survey in Turkey (1, 32). Subsequently, in July 2020, one survey commenced in the United Arab Emirates and two surveys commenced in the United States (33–35). In mid-September 2020, another survey was conducted in the United Kingdom (36). Lastly, another survey was conducted in the United States in October (37). The start date/month of the vaccine survey was not mentioned in the remaining study, conducted in Saudi Arabia but was included in the scoping review (38).

### Conceptual Framework

**Figure 3:**
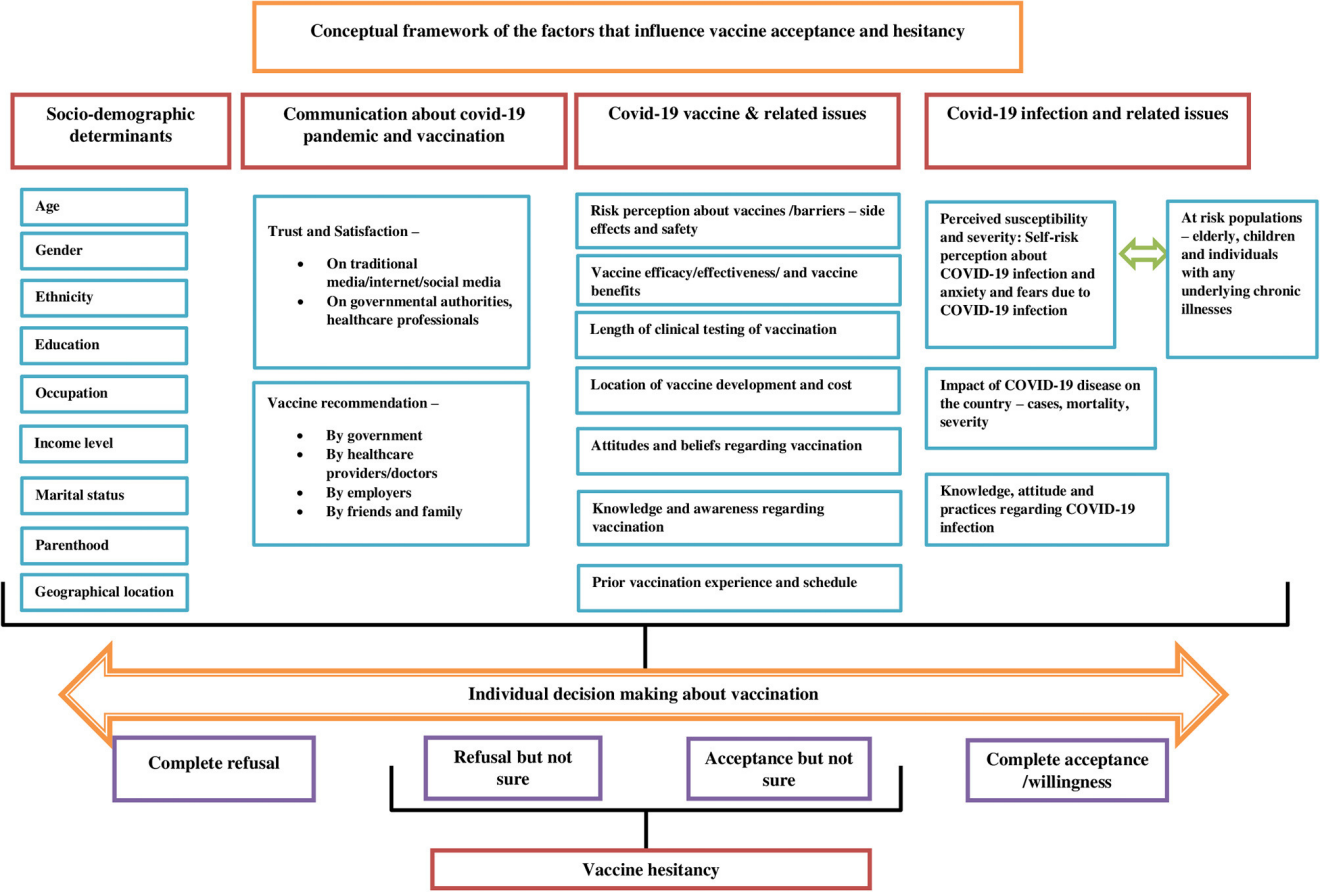
Conceptual framework of the factors that influence vaccine acceptance and hesitancy.

The scoping review presents a conceptual framework of the factors that drive vaccine acceptance and hesitancy (Figure 3). Understanding the barriers and facilitators of vaccines is a crucial step in implementing an effective intervention. The socio-demographic determinants are significantly associated with vaccine acceptance among different population groups. The higher vaccine acceptance was associated with socio-demographic factors such as high income (1, 28), male gender (1, 19, 21, 23, 29, 30), older age (1, 23, 29, 38), married individuals (21, 38), older children with vaccine coverage and no chronic illness (22), high education attainment (29, 31, 35, 38), and health insurance coverage (35). On the other hand, lower vaccine acceptance was associated with factors such as parenthood (19, 31), homemaker (28), retired (20), unemployment (29), the child having a chronic illness (22), younger age (<60 years) (27), black race (27–30), low educational attainment (27, 29, 31), rural settings (27), low income (30, 31) and no health insurance (30). The healthcare workers (HCWs) were observed to be supportive of a COVID-19 vaccine than non-HCWs (20, 30).

The paucity of awareness and knowledge about “who, where, and when” one should be vaccinated and satisfaction with the evidence available on vaccination influences vaccination decisions. Suspicion about safety/potential vaccine harms, efficacy, rushed development, cost, and effectiveness of COVID-19 vaccine were among the main predictors of both vaccine acceptance and vaccine hesitancy (20, 21, 28, 30, 35). Prior experiences with vaccinations and vaccination services can influence forthcoming decisions regarding vaccination. For instance, studies have shown that individuals currently vaccinated against seasonal influenza have a strong inclination to accept a COVID-19 vaccine when available (19, 21, 22). Perceived importance and benefits of vaccination such as protection of high-risk children/individuals with chronic disease or family members and desire to return to normal are well-known determinants of vaccine acceptance (22, 24).

Perceiving COVID-19 infection as a severe problem for the country and/or for self is a strong predictor of vaccine acceptance. Studies have shown that higher perceived susceptibility, the severity of COVID-19 infection, and pandemic were more likely to accept the vaccine (20, 35). For instance, HCW's comprehensive knowledge about COVID-19 and their relatively high awareness regarding the infection may lead them to accept vaccines to protect themselves and their family members (20). Further, some studies have shown that population groups having a history of COVID-19 infection or their relations exposed to it were more likely to accept the COVID-19 vaccination (18, 28, 30, 32).

Tailored and evidence-based health communication is vital in influencing positive health behaviors and gaining the confidence of the individuals. Risk perception regarding the COVID-10 infection, vaccines, and vaccine acceptance is closely linked with trust in health professionals, in government, or in public health institutions. Individuals agreed to accept the vaccine if it is a requirement by their employer (1), clear and consistent communication on the infection and vaccine is provided by government officials regarding the safety and effectiveness of the vaccine (1, 31), or recommended by their doctor or the health professional (1, 19, 30). The frequency of watching, listening, or reading the news suggested increased vaccine acceptance (31). However, the media often exaggerated the risks of vaccination, which can lead to decreased vaccine acceptance among the population groups (24).

To develop effective tailored communication strategies and campaign interventions to promote COVID-19 vaccine adoption, policymakers, health experts, and health communication professionals should first, understand the characteristics of the target audiences/non-adopters (39). The framework takes into account two theories namely, Theory of Planned Behavior (TPB) and The Health Belief Model (HBM). The HBM postulates that the likelihood of an individual adopting specific health behavior is determined by the belief in a personal threat of illness or disease, together with a belief in the effectiveness of the recommended health behavior (35). It targets the six main constructs from HBM, including attitudes toward the perceived threat of infection (a) perceived susceptibility and (b) perceived severity, attitudes regarding perceived expectations of vaccination (c) perceived risk and benefits and (d) perceived barriers, (e) cues to action to vaccinate, and (f) self-efficacy for obtaining vaccinations against COVID-19 infection. The framework illustrates that a range of factors influences vaccine acceptance, and hesitancy. The conceptual framework takes into consideration various health behavior theories and a body of empirical literature focusing on determinants of vaccine acceptance, intention, and hesitancy. It would benefit programs promoting vaccine uptake and adherence from optimizing educational messaging while addressing important individual, socio-cultural, and political barriers enabling millions to receive the benefits of vaccination and ultimately enhancing vaccine acceptance. The TPB suggests that behavior is determined by an intention to carry out the behavior, determined by attitudes toward a COVID-19 vaccine (i.e., its perceived benefit), social norms (i.e., whether valued others support getting a vaccine), and perceived behavioral control (i.e., whether the ability to get the vaccine is within an individual's control) as related to getting a COVID-19 vaccine (35).

From the literature review four main factors were derived to develop the conceptual framework:

a) Socio-demographic determinants
b) Communication about COVID-19 pandemic and vaccination
c) COVID-19 Vaccine and related issues
d) COVID-19 Infection and related issues.

### Description of the Included Studies

A descriptive analysis of the 22 eligible studies included in the scoping review was performed (Table 1). The majority of the studies were from the United States (*n* = 8) followed by the studies in the United Kingdom (*n* = 6). Eighty-two percent of the COVID-19 vaccine survey studies had national geographic coverage and were cross-sectional surveys. One-fourth of the studies utilized the snowball sampling technique while 41% (*n* = 9) did not specifically mention the sampling technique. The average sample size of the COVID-19 vaccine survey studies was 2,253 with a range of 316–13,426. Nearly 37% of the COVID-19 vaccine survey studies had a sample size of <1,000. Results also showed that female participation in these COVID-19 vaccine surveys was higher than males (59 vs. 40%). Seventeen of the 22 COVID-19 vaccine survey studies focused on the general population while only three studies focused on healthcare professionals. The majority of the survey studies assessed vaccine acceptance/willingness (*n* = 15) while all the included studies examined the determinants or factors that would influence the uptake of the COVID-19 vaccine.

**Table 1:** Descriptive analysis of the included studies.

**Study characteristics**	**Study attributes**	**Number of studies (%)**	**Study ID**
COVID-19 vaccine studies	United States (US)	8 (36%)	5, 10, 12, 13, 15, 18, 19, 21
	United Kingdom (UK)	6 (27%)	7, 8, 11, 14, 15, 20
	China	3 (13%)	1, 4, 15
	Other than US, UK and China	10 (45%)	2, 3, 6, 8, 9, 14, 15, 16, 17, 22
Geographical coverage	Global	4 (18%)	5, 8, 14, 15
	National	18 (82%)	1, 2, 3, 4, 6, 7, 9, 10, 11, 12, 13, 16, 17, 18, 19, 20, 21, 22
Study design	Cross-sectional	15 (68%)	1, 3, 4, 5, 6, 7, 9, 10, 11, 13, 14, 16, 17, 21, 22
	Not clearly mentioned	7 (32%)	2, 8, 12, 15, 18, 19, 20
Sampling technique	Random sampling	2 (9%)	4, 15
	Convenience sampling	3 (13%)	7, 13, 18,
	Quota matching	2 (9%)	19, 20
	Snow-ball sampling	5 (23%)	3, 14, 16.17, 22
	Purposive sampling	1 (5%)	1
	Not clearly mentioned	9 (41%)	2, 5, 6, 8, 9, 10, 11, 12, 21
Sample size	Average	2, 432	
	Range	316–13, 426	
	Median	1, 306	
	<1, 000	8 (36%)	1, 6, 7, 12, 16, 19, 21, 22
	1, 000–2, 000	7 (32%)	2, 3, 5, 9, 11, 17, 18,
	>2, 000	7 (32%)	4, 8, 10, 13, 14, 15, 20
Gender	Female	24, 865 (58.67%)	
	Male	17, 284 (40.78%)	
	Others	189 (0.73%)	
Target audience	General population	17 (77%)	2, 3, 4, 8, 9, 10, 12, 13, 14, 15, 16, 17, 18, 19, 20, 21, 22
	Healthcare professionals	3 (13%)	1, 2, 6
	Parents/ caregivers/ guardians	2 (9%)	5, 11
	Vulnerable population(elderly/individuals with chronic respiratory disease)	1 (5%)	7
Vaccine outcomes	Vaccine acceptance/vaccine willingness	15 (68%)	2, 3, 7, 8, 11, 12, 13, 14, 15, 16, 17, 18, 19, 20, 22
	Vaccine hesitancy	14 (64%)	6, 7, 8, 10, 11, 13, 14, 15, 16, 17, 19, 20, 21, 22
	Vaccine intention	6 (27%)	1, 5, 6, 9, 10, 21
	Vaccine determinants	22 (100%)	1, 2, 3, 4, 5, 6, 7, 8, 9, 10, 11, 12, 13, 14, 15, 16, 17, 18, 19, 20, 21, 22
Key considerations	Survey date- not clearly mentioned	2 (9%)	21, 22
	Study platform- not clearly mentioned	4 (18%)	8, 11, 14, 15
	Gender distribution- not clearly mentioned	3 (14%)	2, 5, 8

### Variables Examined in COVID-19 Surveys

Data on demographic variables were assessed in 100% (*n* = 22) of the COVID-19 vaccine studies. Vaccine acceptance, perception regarding COVID-19 infection, and Knowledge and Attitudes toward COVID-19 vaccine (vaccine efficacy, location of development, length of clinical testing) and vaccine history, prior vaccination) were other variables most commonly assessed across these COVID-19 vaccine surveys. Studies also recorded information on variables including knowledge regarding COVID-19 (32%; *n* = 7), COVID-19 related news consumption (41%; *n* = 9), and reasons that can influence vaccine willingness (32%; *n* = 7) (Table 2).

**Table 2:** Variables examined in included COVID-19 surveys studies.

**Study variables**	**Number of studies (%)**	**Study ID**
Demographics	22 (100%)	1, 2, 3, 4, 5, 6, 7, 8, 9, 10, 11, 12, 13, 14, 15, 16, 17, 18, 19, 20, 21, 22
Risk perception of vaccine	4 (18%)	7, 9, 12, 22
Knowledge regarding COVID-19 disease	7 (32%)	3, 12, 13, 14, 17, 20, 21
Vaccine acceptance/vaccine willingness	15 (68%)	2, 3, 7, 8, 11, 12, 13, 14, 15, 16, 17, 18, 19, 20, 22
Vaccine hesitancy	14 (64%)	6, 7, 8, 10, 11, 13, 14, 15, 16, 17, 19, 20, 21, 22
Vaccine intention	6 (27%)	1, 5, 6, 9, 10, 21
Impact of COVID-19 on mental health (disease related anxiety, general anxiety, fears about COVID-19)	8 (36%)	6, 7, 8, 9, 13, 14, 16, 21
COVID-19 related news consumption/ primary source of news information /authentic information/guidelines	9 (41%)	7, 8, 9, 12, 13, 14, 17, 20, 21
Trust In authorities and policy support	8 (36%)	7, 8, 12, 14, 15, 17, 20, 22
Personal relations exposed to COVID-19/encounter with confirmed or suspected COVID-19 patients	6 (27%)	1, 9, 12, 13, 16, 21
Knowledge and attitudes toward COVID-19 vaccine and beliefs (vaccine efficacy, location of development, length of clinical testing, perceived benefits and barriers)	10 (45%)	4, 6, 9, 10, 12, 13, 17, 19, 20, 21
Vaccination of children and child's previous vaccination schedule	5 (23%)	5, 11, 16, 20, 21
Overall health satisfaction	2 (9%)	9, 10
Reasons of vaccine hesitancy/not accepting vaccine	5 (23%)	1, 8, 10, 11, 16
Reasons that can influence vaccine willingness	7 (32%)	4, 7, 11, 13, 15, 16, 17
Risk perception regarding COVID-19 (severity of infections, risk of contracting COVID-19, exaggeration of risk)	14 (64%)	1, 3, 5, 6, 7, 9, 10, 11, 12, 13, 14, 16, 20, 21
Influence of COVID-19 on future vaccine behavior	4 (18%)	1, 6, 7, 11
Reasons to vaccinate the child	3 (14%)	5, 11, 16
Reasons to not vaccinate the child	3(14%)	5, 11, 16
Suffering from any underlying disorder	7 (32%)	1, 5, 6, 7, 9, 12, 16
Adherence to protective measures	4 (18%)	8, 13, 17, 21
Acceptance of prior influenza vaccination/previous vaccinations	3 (14%)	1, 16, 20

### Socio-Demographic Variables Examined in the Included Studies

Most common demographic variables gathered include age (100%; *n* = 22), gender (100%; *n* = 22), education (86%; *n* = 19), and occupation/employment status (64%; *n* = 14). Income (54.5, *n* = 12), location (*n* = 10), and race/ethnicity (*n* = 10) were additional demographic variables examined in the COVID-19 vaccine surveys (Table 3).

**Table 3:** Socio-demographic variables examined in included COVID-19 surveys studies.

**Study variables**	**Attributes**	**Number of studies (%)**	**Study ID**
Demographics	Age group	22 (100%)	1, 2, 3, 4, 5, 6, 7, 8, 9, 10, 11, 12, 13, 14, 15, 16, 17, 18, 19, 20, 21, 22
	Gender	22 (100%)	1, 2, 3, 4, 5, 6, 7, 8, 9, 10, 11, 12, 13, 14, 15, 16, 17, 18, 19, 20, 21, 22
	Marital status	12 (55%)	2, 3, 4, 9, 10, 11, 13, 16, 17, 20, 21, 22
	Education	19 (86%)	2, 3, 4, 5, 7, 8, 9, 10, 12, 13, 14, 15, 16, 17, 18, 19, 20, 21, 22
	Income	12 (55%)	3, 9, 10, 11, 13, 14, 15, 16, 17, 18, 20, 21
	Location/urbanity/city of residence/region	10 (45%)	3, 4, 8, 9, 10, 11, 12, 13, 14, 20
	Race/ethnicity	10 (45%)	9, 10, 11, 12, 13, 14, 18, 19, 20, 21
	Nationality	4 (18%)	14, 17, 19, 22
	Religion	7 (32%)	3, 9, 13, 14, 19, 20, 21
	Occupation/current employment status/private or public sector/working in low risk or high risk setting	14 (64%)	1, 2, 3, 4, 6, 9, 10, 11, 12, 16, 17, 20, 21, 22
	Deprivation category	2 (9%)	7, 13
	Number of family members/household size/family type	3 (14%)	10, 16, 17
	Children/parenthood	6 (27%)	2, 5, 7, 14, 16, 21
	Political affiliation or leaning	6 (27%)	13, 19, 18, 20, 21
	Insurance status/satisfaction with insurance	4 (18%)	13, 16, 19, 21

### Vaccine Acceptance, Vaccine Intention, and Vaccine Hesitancy

Studies recorded information on the variables that influenced COVID-19 vaccine acceptance, vaccine hesitancy, and vaccine intention (Supplementary Table 2).

### Vaccine Acceptance

Five of the 22 studies assessed only vaccine acceptance (19–21, 29, 34). Out of the 22 studies, 16 studies assessed vaccine acceptance and hesitancy (1, 24, 25, 28, 30–32, 35, 36, 38). The studies assessed vaccine acceptance among the respondents by asking a survey question of whether they would be willing to get/accept the new COVID-19 vaccine (yes/no) (1, 20, 21, 28, 29, 31, 32, 35, 36, 38). In a study conducted by Williams et al. vaccine acceptance was measured when the respondents responded to a question of whether they would want to receive a vaccine for coronavirus infection if it becomes available (24). In some studies, vaccine acceptance was measured in terms of how willing respondents would be to get a COVID-19 vaccine if available (25, 30). In a study conducted by Kreps et al. vaccine acceptance was measured by making participants evaluate 2 hypothetical COVID-19 vaccines following which respondents were asked to indicate how likely or unlikely they would be to receive each vaccine individually on a 7-point Likert scale (34). Most common socio-demographic influencers of vaccine acceptance reported in the studies were age group (*n* = 7), gender (*n* = 9), education (*n* = 6), and occupation/employment status (*n* = 6). Trust in authorities (Government, health system, health care providers, and employer) for recommendation and information COVID-19 infection and vaccine had a strong impact on the vaccine acceptance/willingness (*n* = 9). Risk perception of COVID-19 infection was another factor that influenced vaccine acceptance /willingness reported across nine studies. Vaccine efficacy (*n* = 6), vaccine safety (*n* = 11), and prior seasonal vaccination schedule (*n* = 7) were other influencers of vaccine acceptance/willingness.

### Vaccine Intention

Out of the 22 studies, three studies assessed only vaccine intention (18, 22, 26), and three studies assessed vaccine intention in combination with vaccine hesitancy (23, 27, 38). Participants responded about their intention to get the COVID-19 vaccine (yes/no), if available (18, 22, 26). Factors that had an impact on intent to get COVID-19 vaccine across studies included gender [*n* = 3], trust in authorities [*n* = 1], previous seasonal vaccination schedule (*n* = 1), vaccine safety (*n* = 4), and vaccine efficacy (*n* = 4).

### Vaccine Hesitancy

Vaccine hesitancy was measured based on uncertainty regarding vaccine uptake among the study respondents in the included studies. The studies assessing the prevalence of vaccine hesitancy measured the percentage of “not sure” choices answered by the respondents when presented with specific questions related to vaccination (1, 23–25, 27, 28, 30–33, 35–38). Out of the 22 studies, ten studies assessed vaccine hesitancy in combination with vaccine acceptance (1, 24, 25, 28, 30–32, 35, 36, 38). Also, vaccine hesitancy was assessed in combination with vaccine intention in three studies (23, 27, 37). Few variables which had an impact on the vaccine hesitancy across all the studies included age group (*n* = 2), gender (*n* = 3), education (*n* = 1), risk perception of infection (*n* = 3), vaccine safety (*n* = 3), and vaccine efficacy (*n* = 2).

### The Global Prevalence of COVID-19 Vaccine Acceptance, Intention, and Hesitancy Among General Population

**Figure 4:**
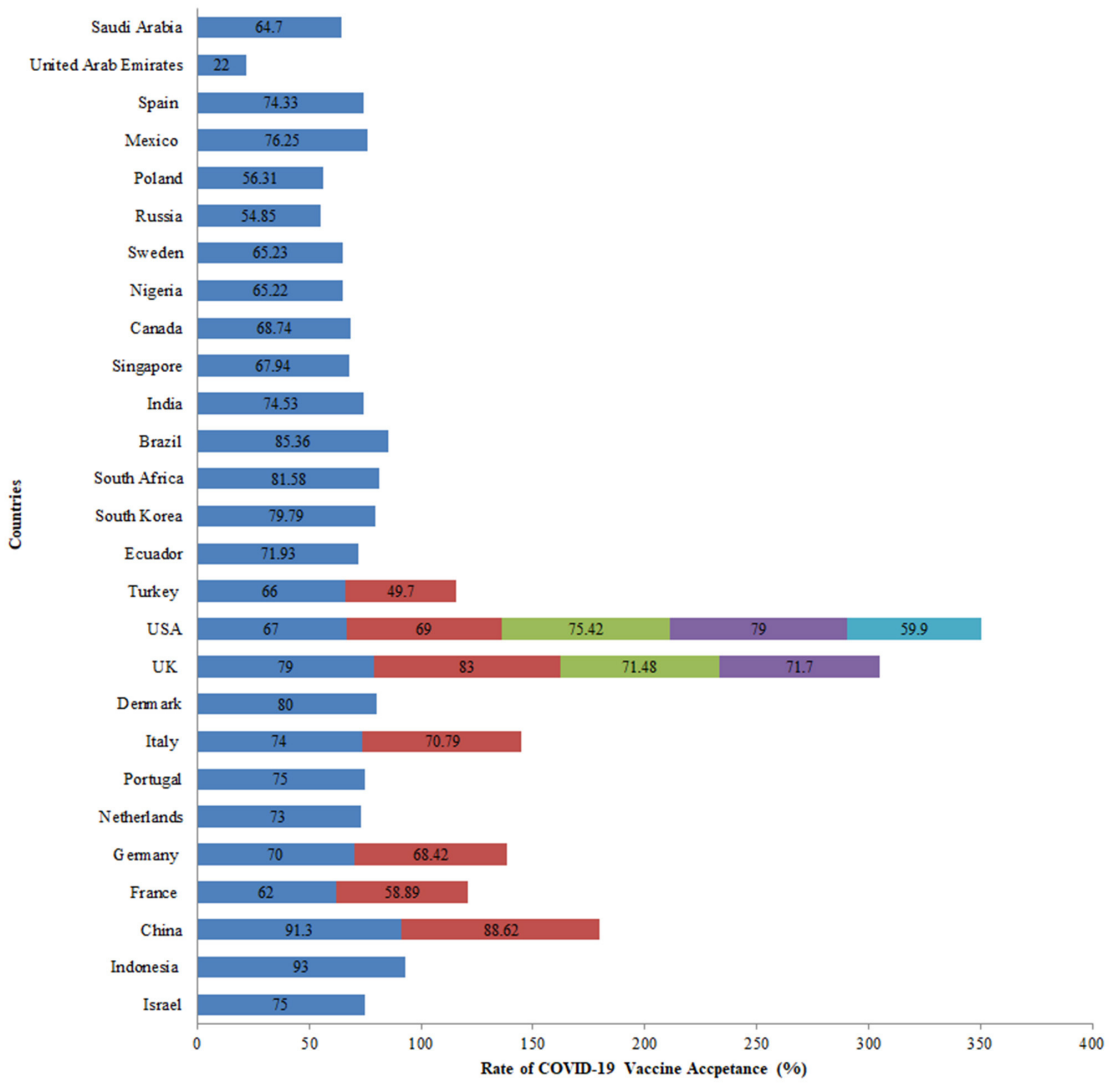
COVID-19 vaccine acceptance rates among the general population across various countries.

**Figure 5:**
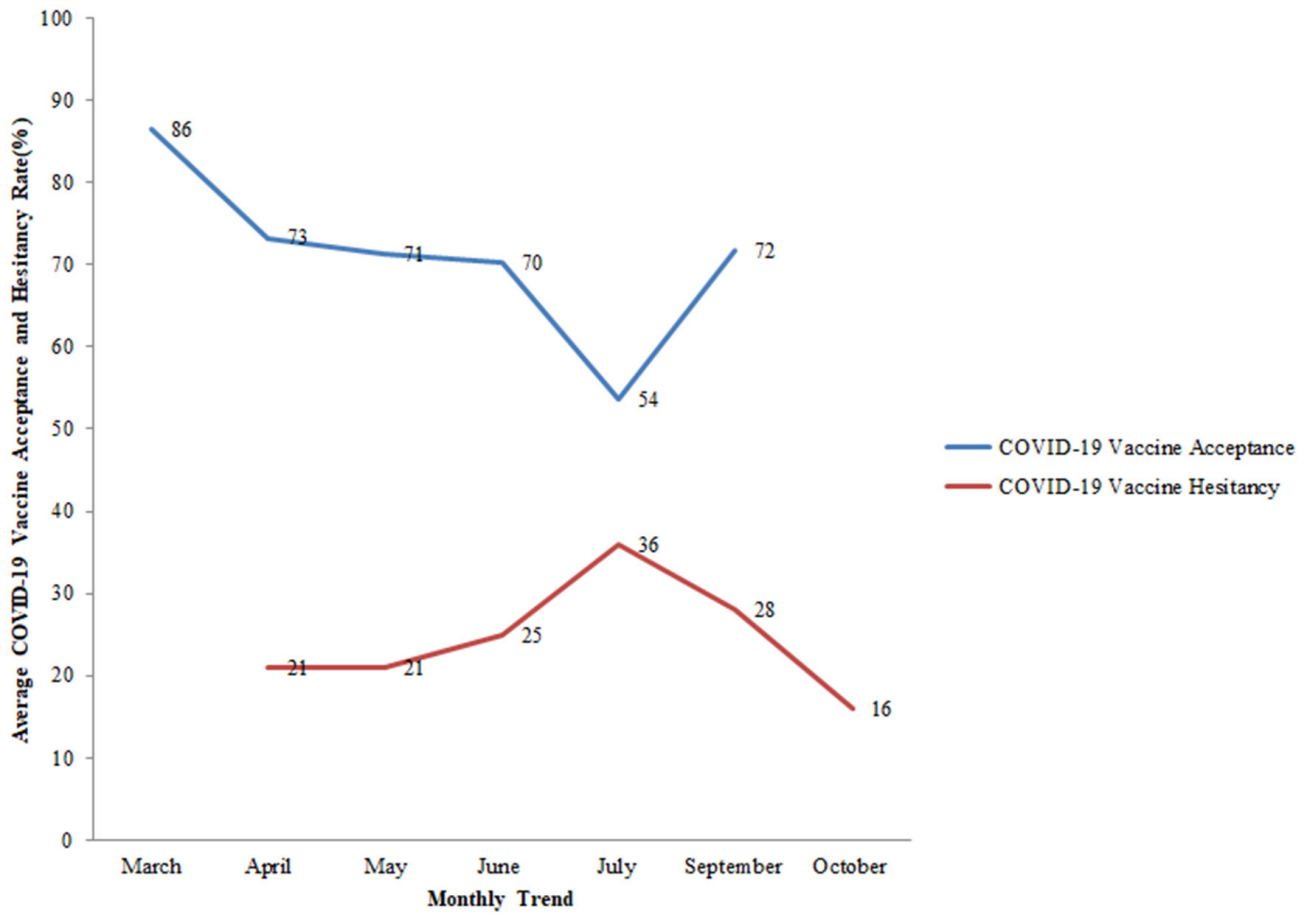
Monthly trend of average COVID-19 vaccine acceptance (%) and average COVID-19 vaccine hesitancy (%) among the general population globally. Study ID 22 not included in the trends as the month of the survey was unknown.

COVID-19 vaccine acceptance was assessed in 27 countries, the majority of which were conducted in the United Kingdom and the United States. Of all the studies related to vaccine acceptance, high acceptance for the COVID-19 vaccine was seen in Indonesia (93%), China (91%), United Kingdom (86%), Brazil (85%), South Africa (82%), Denmark (80%), and South Korea (80%). On the other hand, a comparatively lower vaccine acceptance rate of 22% was observed in the United Arab Emirates. In the USA, COVID-19 acceptance ranged from 60 to 79% among the general population while in the UK, it ranged from 71 to 83% among the general population (Figure 4). Globally, the following changes in COVID-19 vaccine acceptance rates were observed: in March 2020, the average vaccine acceptance observed was 86% which dropped to 54% in July 2020. However, this rate of vaccine acceptance increased to 72% in September 2020 (Figure 5).

**Figure 6:**
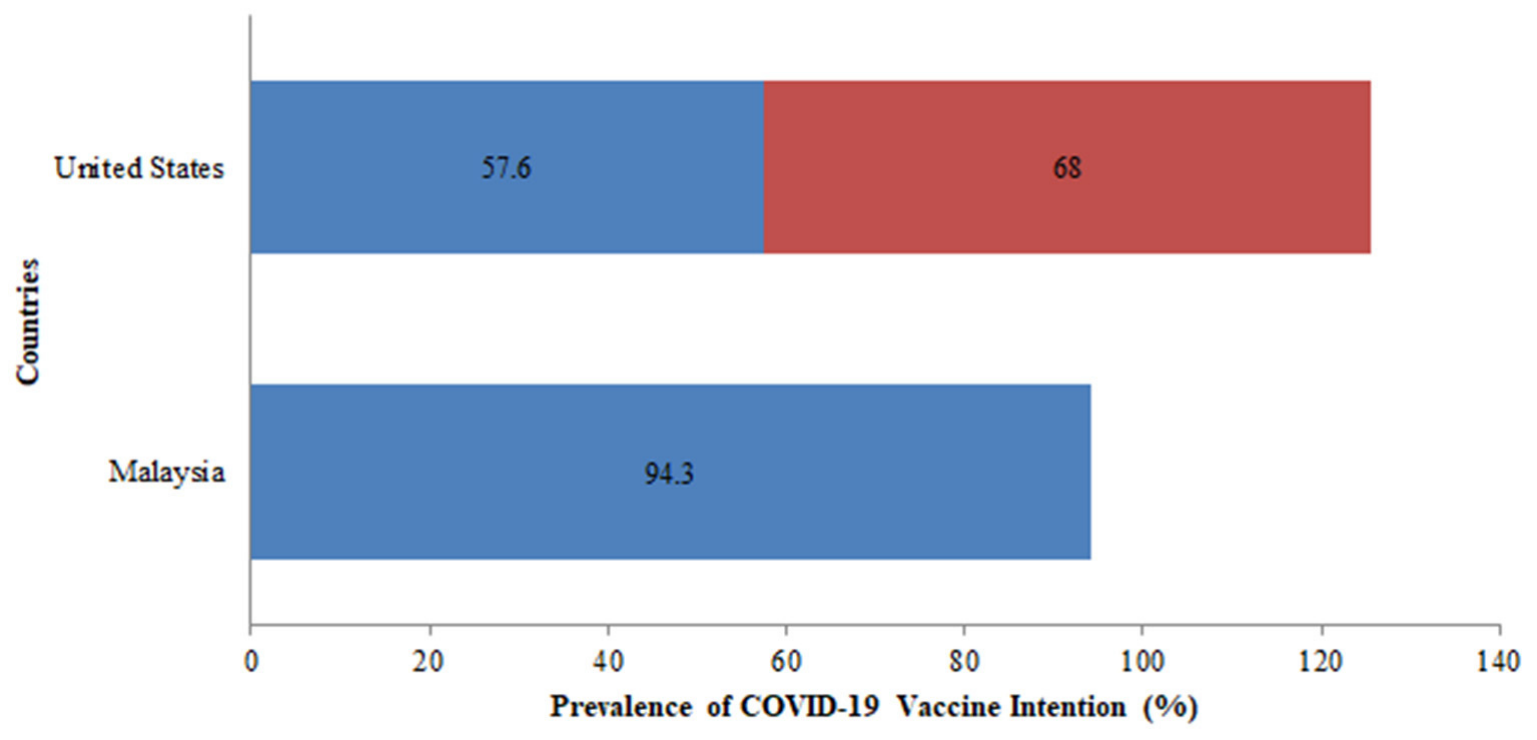
Prevalence of COVID-19 vaccine intention across various countries among the general population.

Additionally, vaccine intention was assessed among the two countries namely, the USA and Malaysia. In the USA, the vaccine intention was more than 50% in the two surveys conducted and the rate of vaccine intention increased from 58% in April 2020 to 68% in October 2020 (Figure 6). There was a slight drop in the rate of vaccine intention among the general population between April 2020 (76%) and October 2020 (68%).

**Figure 7:**
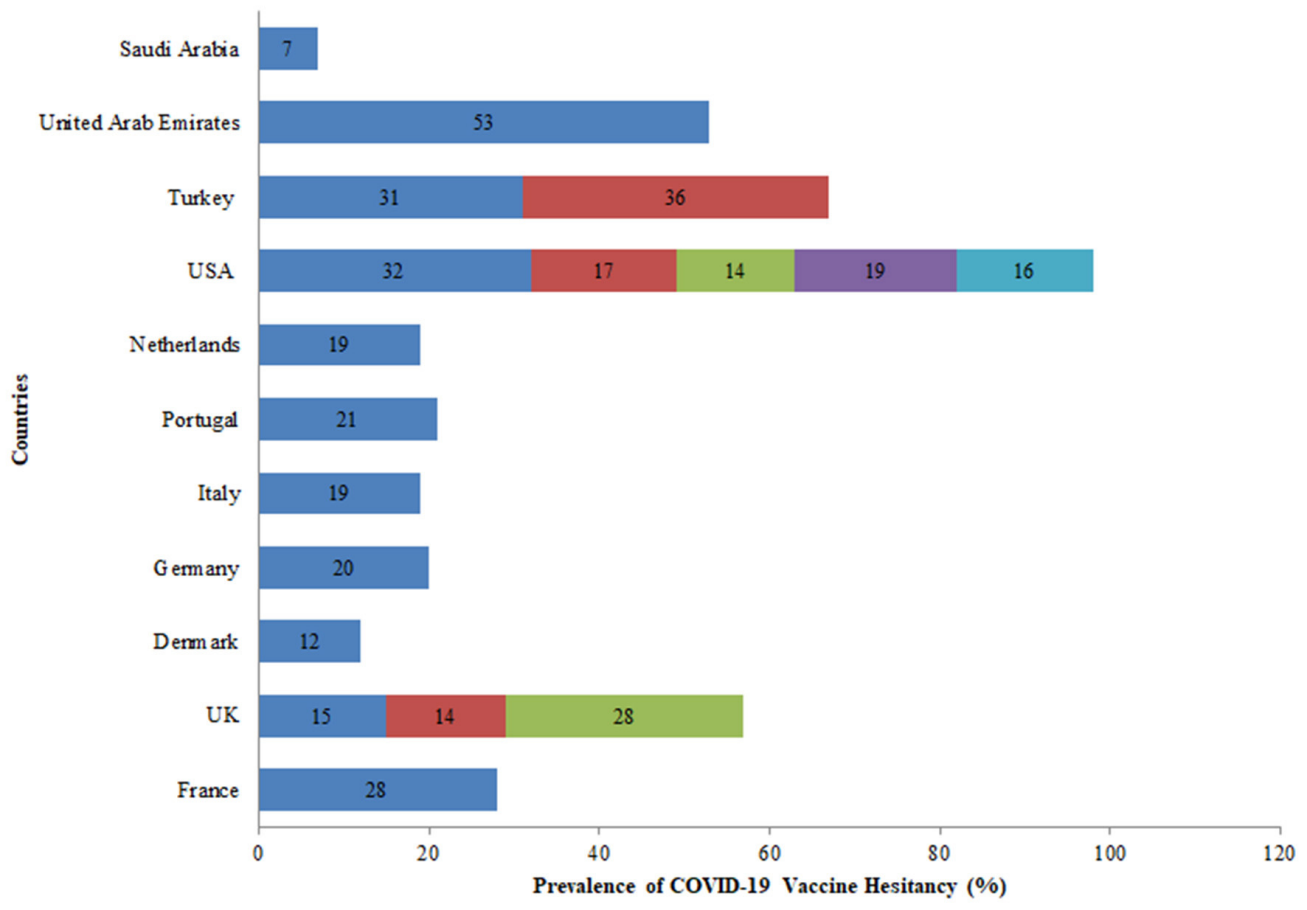
Prevalence of COVID-19 vaccine hesitancy worldwide among the general population.

Further, eleven countries assessed the hesitancy toward the COVID-19 vaccine among the general population out of which five studies were conducted in the USA. In the USA, the rate of vaccine hesitancy gradually decreased over the months. In April 2020, the hesitancy toward getting the COVID-19 vaccine was 32% which declined to 16% in October 2020 among the general population. While in the UK, the rate of COVID-19 vaccine hesitancy increased over time from 15% in April 2020 to 28% in September 2020 among the general population (Figure 7). Globally, the average rate of vaccine hesitancy in April 2020 was 21%, which increased to 36% in July 2020 and later declined to 16% in October 2020 among the general population (Figure 5).

### Socio-Demographic Determinants of COVID-19 Vaccine

**Figure 8:**
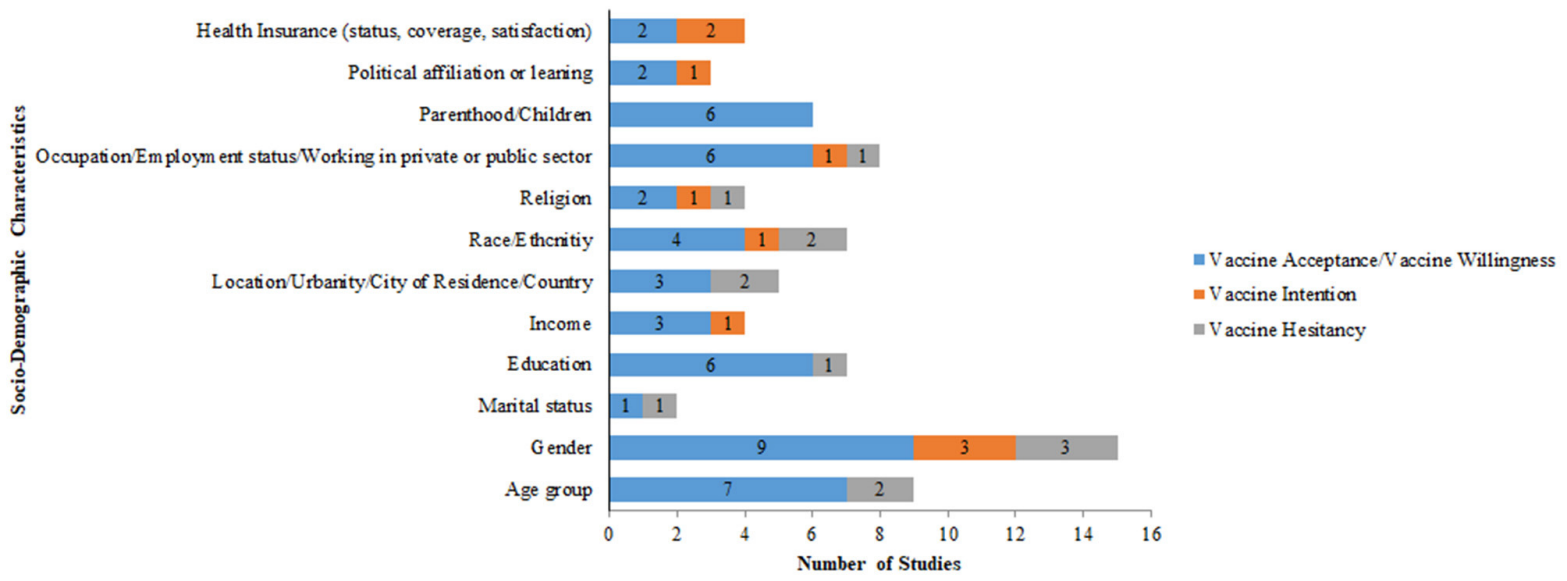
Socio-demographic factors affecting COVID-19 vaccine acceptance, vaccine intention, and vaccine hesitancy.

Figure 8 reports the socio-demographic variables that influence vaccine acceptance, vaccine hesitancy, and vaccine intention. Gender (*n* = 9), age (*n* = 7), education (*n* = 6), occupation/employment status/working in private or public sector (*n* = 6) and parenthood (*n* = 6) were some of the other variables influencing vaccine acceptance/willingness. Gender (*n* = 3) was an important variable influencing vaccine intention and vaccine hesitancy.

### Key Findings of the Included Studies

Table 4 depicts the key findings for each of the reviewed studies in terms of the prevalence of vaccine acceptance/willingness, intention, and/or hesitancy. Variables such as trust in authorities for recommendation and information on safety and effectiveness of COVID-19 Vaccine (*n* = 9), and risk perception of COVID-19 infection (*n* = 8), vaccine efficacy (*n* = 6), current or previous seasonal/influenza vaccination and experiences (*n* = 5), and vaccine safety (*n* = 11) were associated with a higher rate of vaccine acceptance in the included studies. Vaccine safety (*n* = 4) and vaccine efficacy (*n* = 4) were associated with a lower rate of vaccine intention, while risk perception of COVID-19 infection (*n* = 3) and vaccine safety (*n* = 2) were associated with a higher rate of vaccine hesitancy among the participants in the included survey studies. Additional variables influencing vaccine hesitancy included previous vaccination schedule (*n* = 1), and vaccine efficacy (*n* = 2).

**Table 4:** Variables influencing COVID-19 vaccine acceptance, intention, and hesitancy.

**Variable categories**	**Vaccine acceptance/ willingness**	**Vaccine hesitancy**	**Vaccine intention**	**Studies assessing predictors of vaccine acceptance, intention, and hesitancy globally**																					
				**1**	**2**	**3**	**4**	**5**	**6**	**7**	**8**	**9**	**10**	**11**	**12**	**13**	**14**	**15**	**16**	**17**	**18**	**19**	**20**	**21**	**22**
Mental health impact	2		2						VI			VI					VA		VA						
Communication and media	3		2					VI		VA		VI					VA			VA					
Trust in authorities for recommendation and information on safety and effectiveness of COVID-19 vaccine	9	1	1				VI							VA	VA	VA	VA	VA		VA	VA		VH	VI	VA
History or exposure to COVID-19 disease	3		1	VI										VA		VA		VHVA							
Vaccination of children and their previous vaccination schedule			1					VI																	
Risk perception of COVID-19 infection	8	3	3		VA	VA	VA	VI	VI	VA		VI	VH			VA				VA		VA& VH	VH		VA
Subjective norms	2	2														VA				VA		VA& VH	VH		
Vaccine efficacy	6	2	4	VI		VA		VI		VA		VI	VH			VA				VA	VA	VA& VH		VI	
Perceived impact of COVID-19 on country/ Attitude/Belief	4						VA			VA						VA								VI	
Current or previous seasonal/influenza vaccination and experiences	5	1	4	VI	VA		VA	VI	VI				VH		VA				VA	VA				VI	
Vaccine safety (safety, side effects)	11	3	4	VI			VA	VI		VA	VA & VH	VI	VH	VA		VA	VA	VA	VA	VA	VA	VA& VH		VI	
Vaccine development	4	1																		VA	VA	VA& VH		VI	
Anti-vaccine attitudes, beliefs and emotions	2	1	1								VA & VH		VI							VA					
Lifestyle coping	3		2					VI		VA		VI		VA					VA						
Health history	2		2	VI				VI		VA						VA									
Previous or future international travel	1															VA									
Preferred mode of vaccine administration	1																			VA					
Perceived barriers to COVID-19 vaccine uptake- price, travel, time, availability	3	1	2	VI			VI								VA					VA		VA& VH			
The general perception of COVID-19 vaccine	1																			VA					
The general perception of COVID-19 infection and belief of COVID-19 origin	3	1									VA & VH						VA			VA					

*VA, Vaccine acceptance; VI, Vaccine intention; VH, Vaccine hesitancy; VA & VH, vaccine acceptance and vaccine hesitancy*.

### Summarizing Key Findings of the Included Studies According to the Target Population

Table 5 summarizes the key findings of the included studies based on the study population. Some of the common higher associations of vaccine acceptance/willingness were seen among the general population aged 18 years and above were occupations, higher perceived risk of COVID-19 infection, vaccine efficacy, gender (male), marriage status, influenza vaccination history, higher educational attainment, higher COVID-19 anxiety, government satisfaction, age more than 25 years or older ages, Cases and mortality per million of a nation's population, length of vaccine testing. Few concerns of parents/caregivers regarding COVID-19 were a novelty and rapid development of the vaccine, child not perceived to be at risk to contract COVID-19, side effects/safety concerns, and efficacy concerns.

**Table 5:** Detailed key findings of the included studies according to the target population.

**Study ID**	**Target population**	**Key findings**
2, 3, 4, 8, 9, 10, 12, 13, 14, 15, 16, 17, 18, 19, 20, 21, 22	General population	**Higher association of vaccine acceptance:** Being a healthcare worker/occupation, higher perceived risk of COVID-19 infection, vaccine efficacy, gender (male), marriage status, influenza vaccination history, valuing doctor's recommendations, high perception of benefits, low perceived barriers to receiving the vaccine, higher educational attainment, moderate or liberal in their political leaning, belief in Natural origin of the infection, frequency of watching/listening/ reading the news, higher COVID-19 anxiety, government satisfaction, Age more than 25 years or older ages, Cases and mortality per million of a nation's population, history of COVID-19 infection, employer's recommendation for the vaccine, longer protection duration, having insurance, Vaccine offered by the government, availability at the local pharmacy, keen attitude, and eagerness to get vaccinated and length of vaccine testing. **Lower association of vaccine hesitancy:** Being Retired confirmed or suspected cases in local areas, valuing vaccination convenience or vaccine price, Potential side effects of the vaccine, against vaccination in general, and religious reasons, younger age (<60 years), Black race, lower educational attainment, and not having received the influenza vaccine in the prior year, low perceived risk, rural settings, unemployment, no health insurance, conservative in their political leaning, Political attributes (endorsement), rushed vaccine development. **Concerns related to vaccine acceptance:** Potential side effects, the vaccine may not be safe, afraid of injections, believe natural or traditional remedies, need for more information, anti-vaccine attitudes or beliefs, and a lack of trust, the vaccine will serve those who produce this virus, Vaccine conspiracy beliefs.
1, 2, 6	Healthcare workers	**Higher association of vaccine acceptance:** Healthcare nurses in the private sector, nurses with chronic conditions, encountering with suspected or confirmed COVID-19 patients, and prior accepted influenza vaccination in 2019, self-perception of high-risk for severe COVID-19 infection, gender (male), Older age. **Lower association of vaccine acceptance:** Suspicion on efficacy, effectiveness, and safety, believing it unnecessary, and no time to take the vaccine, having a child. **Concerns related to vaccine acceptance:** Quality control, potential side effects, and associated COVID-19 illness.
5, 11	Parents/caregivers/guardians	**Higher association of vaccine acceptance:** Older children, children with no chronic illness, children up-to-date on their vaccination schedule, household Income, the recent history of vaccination against influenza, and caregivers concerned their child had COVID-19 at the time of the survey. **Lower association of vaccine acceptance:** Mothers completing the survey, Child having a chronic illness, ethnicity other than a white, homemaker. **Concerns related to vaccine acceptance:** Novelty, the perceived child is not at risk to contract COVID-19, side effects/safety concerns, efficacy concerns, general vaccine refusal, perceived contraindication, and may vaccinate if more information available/recommended by a healthcare provider, newness and rapid development of the vaccine.
7	Vulnerable population (elderly/individuals with chronic respiratory disease)	**Higher association of vaccine acceptance:** Perception that COVID-19 will persist over time. **Lower association of vaccine acceptance:** Predicting that the media have exaggerated the risk. **Facilitator of vaccine acceptance:** Perceptions of risk to personal health, the severity of COVID-19, and health consequences to others. **Concerns:** Vaccine safety.

### Summarizing Key Findings of the Included Studies According to the Countries

Table 6 summarizes the key findings of the included studies. Some of the common associated factors of vaccine acceptance/willingness seen in the United States were male gender, older adults, higher educational attainment, and higher levels of perceived likelihood to get a COVID-19 infection in the future, perceived severity of COVID-19 infection, vaccine history, and efficacy of the vaccine. Some of the associations leading to lower vaccine acceptance/willingness in the United States rushed vaccine development, Black race, a higher level of perceived potential vaccine harms, unemployment, and not having received the influenza vaccine in the prior year. Few concerns regarding the COVID-19 vaccine seen across the globe were suspicions regarding vaccine safety, potential side effects, efficacy, and rapid development of the COVID-19 vaccine.

**Table 6:** The detailed key finding of included studies: country-wise.

**Study ID**	**Country**	**Target population**	**Key findings**
10, 12, 13, 15, 18, 19, 21	United States	General population	**Association with higher vaccine acceptance**: Gender(male), older adults, higher educational attainment, Healthcare providers would recommend vaccination, moderate or liberal in their political leaning, higher levels of perceived likelihood of getting a COVID-19 infection in the future, perceived severity of COVID-19 infection, perceived effectiveness of a COVID-19 vaccine, Cases, and mortality per million of a nation's population, history of COVID-19 infection, trust in government, high-income levels, employer's recommendation for vaccine, Vaccine efficacy, minor side effects, longer protection duration, high perceived benefits of the vaccine, income levels, vaccine history. **Association with lower vaccine acceptance**: Younger age (<60 years), Black race, lower educational attainment, and not having received the influenza vaccine in the prior year, low perceived risk, rural settings, unemployment, lower-income, no health insurance, conservative in their political leaning, higher level of perceived potential vaccine harms, Rushed vaccine development, Major side effects, vaccine low efficacy, Vaccine developed outside the United States, Political attributes (endorsement), whether FDA EUA approved. **Concerns related to vaccine acceptance:** Vaccine-specific concerns, a need for more information, anti-vaccine attitudes or beliefs, and a lack of trust.
5		Parents/caregivers	**Association with higher vaccine acceptance:** Older children, children with no chronic illness, children up-to-date on their vaccination schedule, the recent history of vaccination against influenza, and caregivers concerned their child had COVID-19 at the time of the survey. **Association with lower vaccine acceptance:** Mothers completing the survey, the child having a chronic illness. **Concerns related to vaccine acceptance:** Novelty, the perceived child is not at risk to contract COVID-19, side effects/safety concerns, efficacy concerns, general vaccine refusal, perceived contraindication, and may vaccinate if more information available/recommended by the healthcare provider.
8, 14, 15, 20	United Kingdom	General population	**Association with higher vaccine acceptance:** Gender(male), age Natural origin, Perceived risk of catching COVID-19, frequency of watching/listening/reading the news, higher COVID-19 anxiety, government satisfaction, higher education, Cases and mortality per million of a nation's population, history of COVID-19 infection, trust in government, high-income levels, employer's recommendation for the vaccine, Vaccine offered by the government, availability at the local pharmacy, keen attitude and eagerness to get vaccinated, taking the vaccine is important, encourages family and friends to get vaccinated. **Association with lower vaccine acceptance**: Potential side effects of the vaccine, against vaccination in general, religious reasons, Lower education, and income levels, Less following of all guidelines, less likelihood of taking a diagnostic test. **Concerns related to vaccine acceptance:** Potential side effects, the vaccine may not be safe, afraid of injections, believe natural, or traditional remedies, vaccine conspiracy beliefs.
11		Parents/caregivers	**Association with higher vaccine acceptance:** Household income. **Association with lower vaccine acceptance:** Ethnicity other than white, homemaker. **Concerns related to vaccine acceptance:** Vaccine safety and effectiveness, newness, and rapid development of the vaccine.
7		Vulnerable population (elderly/individuals with chronic respiratory disease)	**Association with higher vaccine acceptance:** Perception that COVID-19 will persist over time. **Association with lower vaccine acceptance:** Predicting that the media have exaggerated the risk. **Facilitator of vaccine acceptance:** Perceptions of risk to personal health, the severity of COVID-19, and health consequences to others. **Concerns:** Vaccine safety.
4, 15	China	General population	**Association with higher vaccine acceptance:** Age, gender (male), marriage status, risk perception, influenza vaccination history, the belief of COVID-19 vaccine efficacy, valuing doctor's recommendations, Higher levels of education, Cases, and mortality per million of a nation's population, history of COVID-19 infection, trust in government, high-income levels, employer's recommendation for the vaccine. **Association with lower vaccine acceptance:** Confirmed or suspected cases in local areas, valuing vaccination convenience or vaccine price, Lower education, and income levels.
1		Healthcare workers	**Association with higher vaccine acceptance:** Healthcare nurses in the private sector, nurses with chronic conditions, encountering suspected, or confirmed COVID-19 patients, and prior accepted influenza vaccination in 2019. **Reasons for refusal and hesitation for COVID-19 vaccination:** Suspicion on efficacy, effectiveness, and safety, believing it unnecessary, and no time to take the vaccine.
2, 6	Others	Healthcare workers	**Association with higher vaccine acceptance:** Self-perception of high-risk for severe COVID-19 infection, gender (male), influenza vaccination, Older age, fear about COVID-19. **Association with lower vaccine acceptance:** Having a child.
3, 8, 10, 14, 15, 16, 17, 22		General population	**Association with higher vaccine acceptance:** Age, Being a healthcare worker/occupation, married, higher perceived risk of COVID-19 infection, vaccine efficacy, gender(male), High perception of benefits, Natural origin, Perceived risk, of catching COVID-19, frequency of watching/listening/reading the news, higher COVID-19 anxiety, Higher levels of education, Cases and mortality per million of a nation's population, history of COVID-19 infection, trust in government and health system, high-income levels, employer's recommendation for the vaccine, health insurance, anxiety level, having children. **Association with lower vaccine acceptance:** Being Retired, Potential side effects of the vaccine, against vaccination in general, religious reasons, having children, Lower education and income levels, new vaccine, belief that COVID-19 infection is a biological weapon and the vaccine will serve those who produce this virus. **Concerns related to vaccine acceptance:** Afraid of injections believe natural or traditional remedies, Vaccine specific influences, time, and money willing to spend for vaccination.
5		Parents/caregivers	**Association with higher vaccine acceptance:** Older children, children with no chronic illness, children up-to-date on their vaccination schedule, the recent history of vaccination against influenza, and caregivers concerned their child had COVID-19 at the time of the survey. **Association with lower vaccine acceptance:** Mothers completing the survey, the child having a chronic illness. **Concerns related to vaccine acceptance:** Novelty, the perceived child is not at risk to contract COVID-19, side effects/safety concerns, efficacy concerns, general vaccine refusal, perceived contraindication, and may vaccinate if more information available/recommended by a healthcare provider.

## Discussion

The success of attaining herd immunity toward COVID-19 infection among the population largely depends on the uptake of the vaccine. The world was able to overcome the challenge of developing a safe, effective, and affordable vaccine. Nevertheless, the availability of safe and effective COVID-19 vaccines is not sufficient. Planning mass vaccination drives for the citizen involves addressing concerns such as logistics including manufacturing, storage, transportation, cost, and equitable distribution. Besides, the critical component of community acceptance toward COVID-19 vaccination will determine the reach and coverage of the vaccination drives. Increased vaccine hesitancy and lower vaccine acceptance will limit the global efforts to eliminate the pandemic and its consequences.

The current scoping review aimed to examine the prevalence and factors that influence COVID-19 vaccine acceptance, intention, and hesitancy using results of the various COVID-19 vaccine surveys conducted globally.

Five of the 22 studies assessed only vaccine acceptance (19–21, 29, 34). Out of the 22 studies, 16 studies assessed vaccine acceptance and hesitancy (1, 24, 25, 28, 30–32, 35, 36, 38). Out of the 22 studies, three studies assessed only vaccine intention (18, 22, 26), and three studies assessed vaccine intention in combination with vaccine hesitancy (23, 27, 38). The majority of the studies were conducted in the USA (*n* = 8/22) followed by the studies in the UK [*n* = 6/22]. Seventy-seven percent of the included studies focused on the general population while only 13% of the included studies focused on healthcare professionals.

First, in March 2020, the average vaccine acceptance observed globally was 86% which dropped to 54% in July 2020. While in September this rate increased to 72%. Further, eleven countries assessed the hesitancy toward the COVID-19 vaccine among the general population. In the USA, the rate of vaccine hesitancy gradually decreased over the months. In April 2020, the hesitancy toward getting the COVID-19 vaccine was 32% which declined to 16% in October 2020 among the general population. While in the UK, the rate of COVID-19 vaccine hesitancy increased over time. Nineteen percent of participants exhibited vaccine hesitancy in a study conducted in seven European countries (25). Globally, the average rate of vaccine hesitancy in April 2020 was 21%, which increased to 36% in July 2020 and later declined to 16% in October 2020 among the general population.

Large variability in the rates of vaccine acceptance was observed among the general population aged 18 and above across all surveys included in the review. In the USA, COVID-19 acceptance ranged from 60 to 79% in five surveys among the general population (1, 29, 30, 34, 35). While in the UK, it ranged from 71 to 83% in another four surveys among the general population (1, 25, 31, 36). A global survey conducted on willingness to get COVID-19 vaccine reported the acceptance rates as 55% in Russia, 56% in Poland, 59% in France, 65% in Sweden, 65% in Nigeria, 68% in Singapore, 69% in Canada, 71% in the UK, 72% in Ecuador, 74% in Spain, 75% in India, 75% in the USA, 76% in Mexico, 80% in South Korea, 82% in South Africa, 85% in Brazil, and 89% in China (1). A European survey found similar vaccine acceptance rates of 62% in France, 70% in Germany, 73% in the Netherlands, 74% in Italy (excluding Lombardy), 75% in Portugal, 79% in the United Kingdom, and 80% in Denmark (25). In the Middle East, 55 and 65% vaccine acceptance rates were found in UAE and Saudi Arabia, respectively (33, 37). The highest vaccine acceptance rates were found in a second survey conducted in China (91%) and 93% in another survey conducted in Indonesia (20, 21). While the lowest vaccine acceptance rate of 50% was observed in Turkey (32).

The healthcare professionals were given the priority to get the COVID-19 vaccine shot. In our scoping review, a few surveys involved a sample of healthcare workers including doctors and nurses (18, 19, 23). In a survey conducted among nurses in China, about 40% of the respondents intended to accept the COVID-19 vaccine (18) while a higher vaccine acceptance rate was seen among healthcare staff (78% among doctors, and 61% among nurses) in Israel (19). In France, a similar rate of vaccine intention was seen among healthcare workers. About 77% of the respondents intended to get vaccinated against COVID-19 upon its availability (23). The healthcare workers are at constant risk of acquiring an infection while fighting the spread of COVID-19 and thus there is a greater need for protective measures (40–42). The studies in the scoping review demonstrated that recommendations from doctors play a huge role in the increased uptake of the vaccine by the general population. Higher acceptance of vaccines among healthcare professionals can aid in gaining public confidence in the safety of the vaccine (21, 30, 38). Though being a healthcare provider increased the rate of acceptance (18–20) still there a need to educate and create awareness about the COVID-19 vaccine to further enhance vaccine acceptance among healthcare workers. It was seen that during the initial rollout of the COVID-19 vaccine the intent to get vaccinated was <50% (18) which gradually increased as demonstrated in the studies conducted in Israel (19) and France (23).

Further, a different type of population sample involved parents and child-caregivers was utilized in a survey that reported a 65% vaccine intention (22). A comparatively lower vaccine acceptance rate of 56% was observed in a similar population sample in the UK (28). Only one survey focused on older adults and chronic respiratory disease sample which showed a higher vaccine acceptance rate of 86% (24).

Two recent surveys conducted in January 2021 also reveal variability in reporting of these rates of COVID-19 vaccine acceptance, hesitancy, and intention. In Bangladesh, 75% vaccine acceptance is reported among the general population (43). In Turkey, the survey focused on all three types of population samples mentioned in the review which reported a vaccine acceptance rate of 35% among the general population, 52% among healthcare workers, and 29% among patients with rheumatic diseases (44). In India, another interview-based survey carried out between March 2021 and April 2021 showed a 54% vaccine acceptance among patients with systemic autoimmune rheumatic disease. The study also assessed the predictors that affect the vaccine uptake such as age and education (45).

Implementing an effective and full coverage mass vaccination drive would require addressing vaccine hesitancy. To address the vaccine hesitancy it is important to understand the beliefs of the people, the motivation behind getting vaccinated, and factors influencing specific populations to reject the vaccine. The main goal of implementing mass vaccination should be to address the causes of hesitancy through tailored interventions based on the individuals' concerns and parameters. The conceptual framework presented in this study aimed to illustrate a wide range of factors influencing vaccine acceptance and hesitancy. It was framed taking into consideration various health behavior theories and a body of empirical literature focusing on determinants of vaccine acceptance, intention, and hesitancy. The framework would benefit programs promoting vaccine uptake to optimize educational messaging while addressing important individual, socio-cultural, and political barriers. Guidance and recommendations on the issues causing skepticism toward vaccines by the trusted government authorities, health professionals, and public health experts can enable millions to receive the benefits of vaccination, enhance vaccine acceptance and achieve herd immunity.

While the capacity to conclude different factors hampered due to heterogeneity between the included studies, various predictors influencing the COVID-19 vaccine acceptance, hesitancy and intention were identified. Across different geographies, high disparities existed across different socio-demographic characteristics. Old age (23, 38), males (1, 19, 21, 23, 25, 26, 29, 31, 32), individuals with higher education (1, 29, 31, 35, 38), marital status (21, 38), and high income groups (1, 27, 37), were more likely to accept the COVID-19 vaccine.

While factors such as parenthood (19, 22), black race (27–30), no prior vaccination (27), no health insurance (30), and low disease risk (27) decreased the rate of vaccine acceptance. Detailed analyses of the predictors showed lower COVID-19 acceptance vaccine rates among women than men. Since parenthood led to a decrease in vaccine acceptance it is important to gain the trust and confidence of the parents especially the mothers in the COVID-19 vaccine. Parents' hesitancy toward vaccines can pose a difficulty in getting children vaccinated.

Further, COVID-19 disease risk perception (20–24, 26, 30, 31, 35, 38), disease persistence and severity (24, 30), vaccine efficacy and benefits perception (26, 30, 34, 37), prior influenza vaccination (18, 20–23, 37), belief of vaccine efficacy (20, 21), trust in doctor (21, 30, 38), trust in government (1, 36), employer's recommendation (1), confirmed cases and mortality due to COVID-19 (1) were associated with higher COVID-19 vaccine acceptance. These predictors could be utilized for a correct and accurate elucidation of COVID-19 vaccine acceptance, hesitancy, and intention rates among different population groups.

The review reinforces all the possible factors that influence COVID-19 vaccine acceptance and vaccine hesitancy. However, the review is subjected to certain limitations due to the heterogeneity in the study designs, target populations, and study characteristics observed in the included studies that potentially impact the conclusions drawn from the data. The findings of the review have potential implications for further research. It is recommended that future research should elucidate the reasons underlying the association between the studied factors and vaccine uptake. Also, since the included studies evaluated self-reported preferences, they may be subjected to bias as the respondents showing a willingness to get vaccinated might not accept it in reality. Another limitation of the study was the sole dependence on only one search term to find relevant literature which may have led to missing a few relevant studies. However, this approach was utilized to find a broad range of studies focusing on COVID-19 vaccine surveys. The whole purpose of the review was to illustrate a succinct summarization of the prevalence and predictors of the COVID-19 vaccine acceptance and hesitancy worldwide. Further, the latest evidence on the topic used to formulate the discussion section of the review increased the breadth of the literature covered in the scoping review, thus increasing its strength.

The review included COVID-19 vaccine acceptance, intention, and hesitancy surveys done till mid-December 2020. The study can therefore be utilized as a comparative scoping review to assess whether there was any change in the influence and rate of vaccine acceptance and hesitancy after the start of the vaccination drives across the globe.

## Conclusion

In the scoping review, it was seen that there exists a large unevenness in COVID-19 vaccine acceptance, intention, and hesitancy rates across the globe. A low acceptance rate and a high proportion of hesitancy toward the approved and potential COVID-19 vaccines can greatly limit the efforts to control the COVID-19 infection. More surveys should be conducted among healthcare professionals, parents/caregivers, and populations with chronic diseases to assess the rate of vaccine uptake. The conceptual framework developed by analyzing the factors influencing the vaccine acceptance, intention, and hesitancy among various countries will help in implementing a tailored intervention to address the challenge of low vaccine uptake. It would help public health professionals and the government to know about the type of messages and campaigns to be developed to address the challenge of high hesitancy toward the vaccine.

## Author Contributions

AJ conceptualized the research paper and contributed to manuscript writing. AJ, MK, and RK contributed to drafting the paper, data gathering, manuscript writing, data analysis and interpretation, and critical editing. AE-M, DN, and AG contributed to manuscript final reviewing and providing feedback. All authors contributed to the article and approved the submitted version.

## Conflict of Interest

The authors declare that the research was conducted in the absence of any commercial or financial relationships that could be construed as a potential conflict of interest.

## Publisher's Note

All claims expressed in this article are solely those of the authors and do not necessarily represent those of their affiliated organizations, or those of the publisher, the editors and the reviewers. Any product that may be evaluated in this article, or claim that may be made by its manufacturer, is not guaranteed or endorsed by the publisher.
