# Supplementary material for: Predictors of COVID-19 Vaccine Acceptance, Intention, and Hesitancy: A Scoping Review
Source: Front Public Health. 2021 Aug 13;9:698111. doi: 10.3389/fpubh.2021.698111 (PMC8414566; doi:10.3389/fpubh.2021.698111)
Supplement: Supplementary file 1 [file Table_1.DOCX]

**Supplementary Table**

**Table 1. Study ID and title of the included study**

| **Study ID** | **Study title** |
| --- | --- |
| Study 1 | The intention of nurses to accept coronavirus disease 2019 vaccination and change of intention to accept seasonal influenza vaccination during the coronavirus disease 2019 pandemic: A cross-sectional survey (18) |
| Study 2 | Vaccine hesitancy: the next challenge in the fight against COVID‑19 (19) |
| Study 3 | Acceptance of a COVID-19 Vaccine in Southeast Asia: A Cross-Sectional Study in Indonesia (20) |
| Study 4 | Acceptance of COVID-19 Vaccination during the COVID-19 Pandemic in China (21) |
| Study 5 | Caregiver willingness to vaccinate their children against COVID-19: Cross-sectional survey (22) |
| Study 6 | Intention to get vaccinations against COVID-19 in French healthcare workers during the first pandemic wave: a cross-sectional survey (23) |
| Study 7 | Towards intervention development to increase the uptake of COVID-19 vaccination among those at high risk: Outlining evidence-based and theoretically informed future intervention content (24) |
| Study 8 | Once we have it, will we use it? A European survey on willingness to be vaccinated against COVID-19 (25) |
| Study 9 | The use of the health belief model to assess predictors of intent to receive the COVID-19 vaccine and willingness to pay (26) |
| Study 10 | Attitudes Toward a Potential SARS-CoV-2 Vaccine: A Survey of U.S. Adults (27) |
| Study 11 | Parents' and guardians' views on the acceptability of a future COVID-19 vaccine: A multi-methods study in England (28) |
| Study 12 | Determinants of COVID-19 vaccine acceptance in the US (29) |
| Study 13 | Acceptability of a COVID-19 vaccine among adults in the United States: How many people would get vaccinated? (30) |
| Study 14 | COVID-19 vaccine hesitancy is associated with beliefs on the origin of the novel coronavirus in the UK and Turkey (31) |
| Study 15 | A global survey of potential acceptance of a COVID-19 vaccine (1) |
| Study 16 | While studies on the COVID‐19 vaccine is ongoing, the public’s thoughts and attitudes to the future COVID‐19 vaccine (32) |
| Study 17 | Survey data for COVID-19 vaccine preference analysis in the United Arab Emirates (33) |
| Study 18 | Factors Associated With US Adults’ Likelihood of Accepting COVID-19 Vaccination (34) |
| Study 19 | Willingness to get the COVID-19 vaccine with and without emergency use authorization (35) |
| Study 20 | COVID-19 vaccine hesitancy in the UK: the Oxford coronavirus explanations, attitudes, and narratives survey (Oceans) II (36) |
| Study 21 | Influences on Attitudes Regarding Potential COVID-19 Vaccination in the United States (37) |
| Study 22 | Determinants of COVID-19 vaccine acceptance in Saudi Arabia: a web-based national survey (38) |
